# Triple antithrombotic therapy in patients with atrial fibrillation undergoing coronary artery stenting: hovering among bleeding risk, thromboembolic events, and stent thrombosis

**DOI:** 10.1186/1477-9560-10-22

**Published:** 2012-10-18

**Authors:** Mila Menozzi, Andrea Rubboli, Antonio Manari, Rossana De Palma, Roberto Grilli

**Affiliations:** 1 Interventional Cardiology, S. Maria Nuova Hospital; Viale Risorgimento, 80 - 42123 Reggio Emilia, Italy; 2 Division of Cardiology & Cardiac Catheterization Laboratory, Maggiore HospitalLargo Nigrisoli, 2 – 40133, Bologna, Italy; 3 Regional Agency for Health and Social Care, Viale Aldo Moro, 21 - 40127, Bologna, Italy

**Keywords:** Atrial fibrillation, Percutaneous coronary intervention, Stent, Warfarin, Antiplatelet drugs

## Abstract

Dual antiplatelet treatment with aspirin and clopidogrel is the antithrombotic treatment recommended after an acute coronary syndrome and/or coronary artery stenting. The evidence for optimal antiplatelet therapy for patients, in whom long-term treatment oral anticoagulation is mandatory, is however scarce. To evaluate the safety and efficacy of the various antithrombotic strategies adopted in this population, we reviewed the available evidence on the management of patients receiving oral anticoagulation, such as a vitamin-k-antagonists, referred for coronary artery stenting.

Atrial fibrillation is the most frequent indication for oral anticoagulation. The need of starting antiplatelet therapy in this clinical scenario raises concerns about the combination to choose: triple therapy with warfarin, aspirin, and a thienopyridine being the most frequent and advised. The safety of this regimen appeared suboptimal because of an increased risk in hemorrhagic complications. On the other hand, the combination of oral anticoagulation and an antiplatelet agent is suboptimal in preventing thromboembolic events and stent thrombosis; dual antiplatelet therapy may be considered only when a high hemorrhagic risk and low thromboembolic risk are perceived. Indeed, the need for prolonged multiple-drug antithrombotic therapy increases the bleeding risks when drug eluting stents are used.

Since current evidence derives mainly from small, single-center and retrospective studies, large-scale prospective multicenter studies are urgently needed.

## Introduction

Long-term oral anticoagulation (OAC) with vitamin K antagonists is recommended in patients with atrial fibrillation (AF) at moderate to high risk of stroke, those with prosthetic heart valves, previous cardiogenic thromboembolism, recent deep vein thrombosis or pulmonary embolism [1-3].

In the event that also an indication for dual antiplatelet therapy (DAPT) arises, mainly because of percutaneous coronary intervention with stent implantation (PCI-S) [4], the management of these patients becomes challenging. It is estimated that about 5% of patients undergoing percutaneous coronary interventions (PCI) has atrial fibrillation or other indications for chronic oral anticoagulant therapy [5]. Since DAPT provides insufficient protection against stroke and thrombotic complications in patients with AF and mechanical aortic valves [6,7], substitution of OAC with DAPT appears not applicable. The issue therefore, is whether or not aspirin and/or clopidogrel should be added to OAC in patients who are referred for PCI-S.

To date, there are no large-scale studies or randomised clinical trials involving this kind of patients, and their management is often left to physician’s discretion. Triple therapy (TT) with OAC, aspirin and clopidogrel is reported to be the antithrombotic treatment most frequently prescribed over the medium term after PCI-S in patients with an indication for OAC [8].

In this paper we review the evidence supporting the preferential use of TT in AF patients who are submitted to PCI-S. Since the data available for patients with other indications for OAC, such as prosthetic heart valves or pulmonary embolism are extremely limited, these indications will not be covered.

### Available evidence

Various combinations of key words such as oral anticoagulation, warfarin, antiplatelet agents, percutaneous coronary intervention, stent, were used for Medline search.

All English language full-text articles, from January 2004 to December 2011, reporting safety outcomes during follow up in patients with indication for OAC treated with PCI-S were selected for this review. At present, 28 full reports on the safety and efficacy at follow up of antithrombotic treatment in patients on OAC undergoing PCI-S have been published [9-36]. These papers, some of which have been recently meta-analyzed [37], are summarized in Table 1. Although only seven of them focused on AF patients [13,19,20,22,25,29,36], AF was the prominent indication for OAC in most of the studies, so that considerations for clinical practice arising from these studies can be extrapolated to the specific subset of patients with AF.

**Table 1:** Studies published in peer-reviewed journals on the antithrombotic treatment of patients on OAC undergoing PCI-S

**Author**	***Design of the study***	***Enrolment Years***	***Publication Year***	***Patients, n***	***Prevalence of AF in TT, %***	***DES, %***	***TT at discharge, %***	***Outcome comparisons, n***	***Follow up, months***
Orford JL et al. [9]	R/SC	2000-2002	2004	66	39	0	100	None	1
Mattichak SJ et al. [10]	R/SC	2001	2005	40	43	0	100	Against contemporary patients on DAPT, 42	12
Khurram Z et al. [11]	R/SC	NR	2006	107	70	75	100	Against contemporary patients on DAPT, 107	7
Porter A et al. [12]	R/SC	2000-2004	2006	180	37	0	100	None	15
Lip GYH & Karpha M [13]	R/SC	2000-2005	2006	35	100	14	17	Within population	1
Karjalainen PP et al. [14]	R/MC	2003-2004	2007	239	70	42	48	Within population and against contemporary patients on DAPT, 239	12
DeEugenio D et al. [15]	R/SC	2000-2005	2007	97	60	30	100	Against contemporary patients on DAPT, 97	6
Rubboli A et al. [16]	R/SC	2002-2004	2007	49	60	0	41	Within population	1
Nguyen MC et al. [17]	P/MC^	1999-2006	2007	580	40	16	79	Within population	6
Rogacka RCA et al. [18]	R/SC	1999-2006	2008	127	59	56	100	None	21
Ruiz-Nodar JM et al. [19]	R/SC	2001-2006	2008	213	100	40	50	Within population	20
M.-Fernandez S et al. [20]	R/SC	2002-2006	2008	104	100	66	49	Within population	12
Sarafoff N et al. [21]	P/SC	2002-2007	2008	515	78	100	59	Within population	24
Maegdefessel L et al. [22]	R/SC	1999-2004	2008	159	100	NR	9	Within population	16
Rossini R et al. [23]	P/MC	2005-2006	2008	102	67	47	100	Against contemporary patients on DAPT, 102	18
Valencia J et al. [24]	*P/SC*	2004-2006	2008	70	68	60	64	Within population	12
Gao F et al. [25]	P/SC	2005-2008	2010	622	100	100	23	Within population	12
Sambola A et al. [26]	P/MC	2003-2006	2009	405	67	46	68	Within population	6
Gilard M et al. [27]	P/MC	2005-2006	2009	359	48	30	35	Within population	12
Hälg C et al. [28]	R/MC	2003-2004	2009	813	NR	65.9	5	Within population	36
Halbfass P et al. [29]	R/MC	2002-2006	2009	117	100	47	45	Within population	28
Helft G et al. [30]	P/MC	2006-2007	2009	50	62	14	100	None	1
Olson KL et al. [31]	R/SC	2003-2006	2009	175	23	81	100	Against contemporary patients on DAPT, 339	12
Baber J et al. [32]	R/SC	2003-2007	2009	454	53	100	100*	None*	
Persson J et al. [33]	P/MC^	1997-2005	2010	1183	35	32	56	Within population	12
Uchidab Y et al. [34]	R/SC	2004-2007	2010	575	5	100	9	Within population	15
Guasch E et al. [35]	R/MC	2005-2006	2011	33	29	NR	100	Against same patients after stopping clopidogrel	922 pt/months
Jang SW et al. [36]	R/MC	2005-2007	2011	362	100	91	20	Within population	615

OAC= oral anticoagulation; PCI-S= percutaneous coronary intervention with stent implantation; AF= atrial fibrillation;

DES= drug eluting stent; TT= triple therapy; R= retrospective study; P= prospective study; SC= single center; MC= multi center; DAPT= dual anti-platelet treatment; NR= not reported. ^ post-hoc * evaluation of conventional TT vs modified TT (see text).

None of the studies was randomized. Eleven studies specifically assessed the safety of TT of warfarin, aspirin and a thienopyridine, and either no comparisons, or comparisons with contemporary populations with no indications for OAC, receiving DAPT [9-12,15,18,23,30-32,35]. In the remaining studies, the outcomes with different treatments strategies adopted were compared within the population examined [13,14,16,17,19-22,24-29,33,34,36]. Finally, a wide variation in the length of follow-up and in the definitions of the outcome measures was observed making their comparison difficult.

Orford et al. [9] evaluated the safety of 30-day TT in 66 patients after PCI. Bleeding complications occurred in 6 patients (9.2%). In 2 patients (3.1%), the hemorrhagic event was a melena and was classified as major, since blood transfusions were required. In both patients, the INR value at the time of bleeding was above the therapeutic level (12.4 and 3.7, respectively). Minor hemorrhages consisted of a groin hematoma, minor and self-limiting nose and ear bleedings, and a gross hematuria, which were all treated conservatively with temporary withdrawal of one or more antithrombotic agents. No major cardiac events were observed.

Mattichak et al. [10] examined 40 patients with ST-elevation acute myocardial infarction treated with primary PCI-S, and discharged on TT , and compared the outcome to 42 contemporary primary PCI-S patients with no indications for OAC. At 12 months, the occurrence of gastrointestinal bleedings with TT was high, although not significantly different from the control group with DAPT (15% vs. 9%; p=0.12). Also the need for blood transfusions was higher with TT at 12 months (21% vs. 3.5%, p=0.028).

Khurram et al. [11] evaluated 107 patients who were treated up to 12 months with TT following PCI-S (drug-eluting stents in 50% of cases). A contemporary cohort of an additional 107 patients with no indication for OAC who were discharged on DAPT after implantation of drug eluting stents (DESs), served for comparison. The incidence of both major and minor hemorrhages was significantly higher in the TT group than in the control population (6.6% vs. 0%, p=0.014, and 14.9% vs. 3.8%, p=0.01, respectively). At multivariable analysis, TT was found associated to an about 5-fold increase in hemorrhages as compared to DAPT (hazard ratio 5.44; 95% confidence intervals 2.03-14.53 p=0.001). All major bleedings occurred between 2 and 10 months, suggesting that the duration of the three-drug regimen should be minimized, e.g. by avoiding the use of DESs. One of the major hemorrhagic complications was fatal intracranial hemorrhage in a patient with a history of intracranial bleeding. Neither stent thrombosis nor thromboembolic events were observed during the triple antithrombotic therapy.

In the study by Porter et al. [12], 180 patients undergoing PCI-S and discharged on TT were evaluated. AF was the indication for VKA only in 37% of the patients. At 30 days, bleeding complications occurred in 20 patients (11%), being classified as major (i.e., major groin haematoma) in 2 of them (1%). Both major hemorrhagic events occurred during the initial phase of warfarin and heparin overlap. This period appears to be fragile since 18 out of the 20 bleedings observed took place during the bridging period. Neither stent thrombosis nor thromboembolism were observed in this study.

Lip and Karpha [13] included in their small study only patients with AF. At discharge, the prescribed antithrombotic treatment in the 35 patients included was highly variable: DAPT was given in 71% of cases, TT in 17%, clopidogrel alone in 6%, and the combination of warfarin and one antiplatelet agent only (either aspirin or clopidogrel) in the remaining 6%. At 30 days, no bleeding complications requiring hospitalization were observed.

Karjalainen et al. [14] evaluated a population of patients with an indication for OAC undergoing PCI-S at six Finnish hospitals. These 239 patients were then compared with an age- and sex-matched control group with no indication for OAC and discharged on DAPT after PCI-S. The antithrombotic regimens prescribed in these patients were found highly variable: TT in 48% of cases, warfarin and clopidogrel in 21%, aspirin and clopidogrel in 15.5%, warfarin and aspirin in 15%, and warfarin alone in 0.5%. At 12 months, stent thrombosis was significantly more frequent in the warfarin plus aspirin group (15.2%), whereas the lowest incidence was observed with TT (1,9%) (p= 0.004). Occurrence of stroke was also different between subgroups, being more frequent in patients receiving DAPT (8.8% vs. 2.8% with TT) (p= n.s.). Conversely, no significant differences were observed between treatment subgroups in major bleedings. Overall, the OAC group showed a 12-month mortality (8.7% vs. 1.8%; p=0.003) and myocardial infarction (10% vs. 4.8%; p= 0.041) rates significantly higher than the DAPT group, although higher risk baseline features need to be acknowledged.

DeEugenio et al. [15] examined the safety and efficacy of TT in 97 patients undergoing PCI-S or brachytherapy. A matched control group of 97 patients on DAPT and no indication for OAC served for comparison. At 6 months, major bleedings were significantly more frequent in the TT group: 14% vs. 3% (hazard ratio 5.0; 95% confidence intervals 1.40-17.80; p=0.012), and mostly were gastrointestinal in location. Results of multivariate analysis revealed that the risk of major bleeding after PCI was 5 times higher for patients receiving TT compared with DAPT. The number needed to harm was 9 additional patients treated with TT to cause one major bleeding.

Rubboli et al. [16] evaluated 49 consecutive patients with an indication for OAC undergoing PCI-S. The antithrombotic regimens prescribed at discharge in these patients was widely variable: aspirin and either ticlopidine or clopidogrel in 45% of cases, TT with OAC (or low-molecular-weight heparin), aspirin and a thienopyridine in 41%, and the combination of warfarin and aspirin in 14%. At follow up of about 30 days, TT was associated to a higher (albeit not statistically significant) occurrence of bleeding complications: 20% vs. 4.5% (HR 5.25) compared to DAPT. Out of the 4 haemorrhages observed in patients on the three-drug regimen, 3 were major, as opposed to none in the aspirin plus thienopyridine group. Of note, all major bleeding complications occurring in the TT group were observed in the setting of emergency PCI-S for ST-elevation myocardial infarction, and in patients either older than 75 years or having received glycoprotein IIb/IIIa inhibitors or having undergone some traumatic manoeuvre. In one patient out of the 7 receiving the combination of warfarin and aspirin (14%) a subacute stent thrombosis requiring emergency reintervention was observed.

Nguyen et al. [17] evaluated 580 patients who underwent PCI-S for acute coronary syndromes and were discharged on warfarin plus single (220) or dual (580) antiplatelet agents as part of the GRACE (Global Registry of Acute Coronary Events) registry. A significant reduction in stroke was observed in patients receiving TT at 6 months (0.7% vs. 3.4%, p= 0.02). No differences in major bleeding events were observed in-hospital between patients receiving TT (5.9%) or those treated with warfarin and a single antiplatelet agent (4.6%) (p =0.46). No follow-up data on bleeding events were presented.

A series of 127 patients [18] receiving warfarin who were treated with PCI-S and discharged on TT demonstrated a 7.1% total bleeding incidence (4.7% major) during 21-month follow-up. Of note, major bleeding events were fatal in 3 (50%) cases and were intracranial in 4 (approximately 66%) patients. These frequencies should, of course, be interpreted cautiously in light of the small number of events.

In the study by Ruiz-Nodar et al. [19] including 426 patients with AF who underwent PCI-S, TT was administered to 213 (50%) patients. Adverse events were frequent (35%) in the whole population: major bleeding being reported in 12.3%, and all-cause mortality in 22.6% of patients. Among the patients who were prescribed OAC at discharge, there was a non-significant increase in major bleeding (14.9% vs. 9.0%) but a significantly better MACE-free prognosis as well as lower all-cause mortality. Interruption of OAC after intervention was associated with an increased risk of cardiovascular events at a mean of 595 days (HR: 4.9, 95% CI: 2.17 to 11.09; p <0.01) and was largely driven by thromboembolic complications. The clinical benefit of continuing warfarin outweighed a 66% relative increase in risk of major bleeding.

In 104 patients with AF undergoing PCI-S studied by the same group [20], TT was the most commonly used regimen (49%). In this report the authors differentiated between early (≤48 hours) and late (>48 hours) major bleedings. A high rate of late major bleeding was observed in patients receiving TT (21.6% vs. 3.8% in non-TT, p=0.006). At multivariable analysis use glycoprotein II/IIIa inhibitor use and multivessel/left main PCI-S were independent predictors of early major bleedings; while TT use, occurrence of early major bleeding, and baseline anemia were independent predictors of late major bleedings.

The study by Sarafoff et al. [21] was the first that prospectively analysed the safety and efficacy of TT compared to DAPT in a population of patients on chronic OAC all treated with DES. They used clinical and echocardiographic criteria to select patients who continued OAC (306 patients discharged with TT), in the other group patients discontinued OAC for the time they received DAPT (209 patients). The primary endpoint, a composite of death, myocardial infarction, stent thrombosis or stroke, was less frequent in TT group (4.2% vs. 7.2%) respect to DAPT, and also at two years of follow up (14.1% vs. 18%), even though not statistically significant. At two years, the incidence of major bleeding was more frequent in DAPT group (3.1% vs. 1.4%, p= n.s.). The authors concluded that both TT and DAPT were associated with favourable safety and efficacy when the post-procedural treatment was defined by clinical and echocardiographic criteria.

In a German study including 159 patients with AF undergoing PCI [22], Maegdefessel at al. compared patients treated with either DAPT, a combination of DAPT and low-molecular weight heparin, and TT. In a median follow up of 1,4 years patients in DAPT experienced the highest incidence of bleeding events (2 cases vs. 0), myocardial infarction (4 cases vs. 0), stroke (9 cases vs. 4 in group 2 and 0 in group 3), and cardiovascular death (3 cases vs. 5 in group 2, and 1 in group 3).

A prospective multicenter study performed at three Italian institutions [23] enrolled 102 consecutive patients requiring OAC undergoing PCI-S, compared with a control group of 102 patients treated with DAPT. At 18 months, a non significant increase in bleeding was observed in the TT compared to the DAPT group (10.8% vs. 4.9%, p= 0,1). International Normalized Ratio (INR) values were significantly higher in patients with bleeding (2.8 ± 1.1 vs. 2.3 ± 0.2, p =0.0001). An INR >2.6 was the only independent predictor of bleeding. There were no significant differences in major adverse cardiac events between the two groups (5.8% vs. 4.9%, p =0.7).

In the study by Valencia et al. [24], 70 patients on OAC treated with PCI-S were discharged either on TT (64,2%), OAC plus clopidogrel (7,5%), OAC plus aspirin (3%), or DAPT (25.4%). Adverse events were minor bleeding in 11.4% of cases, major bleeding in 8.6%, myocardial infarction 4.3%, stent thrombosis in 1.4%, and death in 12.8%. Patients treated with TT were at increased risk of bleeding complications.

In the prospective study by Gao et al. [25], 622 consecutive patients with atrial fibrillation underwent PCI-S with DES. They were discharged on TT, DAPT, or the combination of warfarin and a single antiplatelet agent. At 12-month follow up, the combined incidence of death, myocardial infarction, target vessel revascularization, stent thrombosis, and stroke was in favour of TT. However, in the TT group, respect to the combination of warfarin plus a single antiplatelet agent and DAPT, a significantly higher incidence of overall bleeding (11.8% vs. 7.4% vs. 5.1%, P=0.038), essentially driven by minor bleeding events (8.8% vs. 5.0% vs. 3.3%, P=0.042), was observed.

A prospective multicenter registry, the MUSICA study, included 405 patients from nine hospitals in Spain and one in UK [26]. TT was administered to 68.6% of patients, OAC plus an antiplatelet agent to 11.4%, and DAPT to 20%. At 6 months, patients in TT group experienced a higher rate of bleeding (15% vs. 13% in patients receiving OAC plus an antiplatelet agent, and 3.7% in patients receiving DAPT; p=0.03). Even though, there were no differences in the incidence of major bleedings among groups. The combination of OAC plus an antiplatelet agent showed the worst rate of adverse events in the whole cohort, especially in patients at moderate–high thromboembolic risk.

In the STENTICO Registry [27] a total of 359 patients treated with PCI with an indication for long-term OAC from 40 French centers were included. In 234 patients (65.2%) OAC therapy was discontinued and DAPT was administered for about 30 days. One hundred and twenty five patients were treated with TT (34.8%). The stroke rate did not differ significantly between DAPT (3.0%) and TT (0.8%) groups. Severe and moderate bleeding, according to the GUSTO criteria, occurred in 2.1% of patients in DAPT and in 6.4% of patients in TT (p =0.04). A significant difference in bleeding rate was found between the femoral and radial approaches (10.3% vs. 3.8%, p =0.01). The authors concluded that, adding DAPT to pre-existing OAC therapy increases the post-PCI bleeding risk. Temporary discontinuation decreased this bleeding risk but tended to increase the risk of stroke. A radial approach for PCI could be a good alternative to avoid bleeding.

In the *post-hoc* analysis of the BASKET trial [28] 44 patients on OAC were considered (5.4% of the whole population). After stenting, all patients were assigned to DAPT in addition to OAC for at least 6 months, and were followed for 3 years. Overall, there were 25 early (i.e. during the index hospitalization) bleeding events (during warfarin therapy in 2 cases), and 26 late bleeding events (during warfarin therapy in 8 cases). Most early bleedings were directly related to the intervention. The most important risk factor for total bleedings was antithrombotic regimen. In patients treated with TT the total bleeding rate was considerably higher (OR= 4.6), resulting mainly from late bleedings (OR= 9.3). The annual rate of bleedings was 6.1% in patients taking warfarin, only 0,8% in those not taking it.

In the retrospective analysis by Halbfass et al. [29], 117 patients with atrial fibrillation treated with PCI-S were evaluated. Fifty-three patients (45.3%) received TT after PCI. Two out of 13 patients (15%) with a major bleeding were on DAPT, 9 out of 13 (69%) were on OAC, but only one patient (8%) was on TT while the bleeding occurred. Only two out of six patients with a thromboembolic complication were on OAC at the time of the occurrence. The probability of the occurrence of adverse events in patients on TT vs. patients without TT after PCI-S was similar (p= n.s.).

In a small France study [30], 50 consecutive patients underwent PCI-S without interrupting oral anticoagulant therapy, and were discharged on TT. No thrombotic events or excess bleeding were observed at 1 month. Only one patient had a minor hemorrhage 8 days after procedure.

In a retrospective study on PCI-s patients [31], Olson et al. evaluated 175 patients treated with TT matched with 339 patients treated with DAPT. There were 25 (14.3%) major hemorrhages in the TT group and 10 (3.0%) major hemorrhages in the DAPT group (OR 9.0; 95% CI, 3.1–26.1). Patients in the TT group had a greater likelihood of MACE compared to patients in the DAPT group (OR 2.0; 95% CI 1.1–3.8). Post-stent treatment with TT was associated with a substantially greater likelihood of major events than treatment DAPT.

In the study by Baber et al. [32], 454 consecutive patients who underwent PCI-S with DES were discharged on TT either conventional (n= 170) or with modified antiplatelet regimen (daily aspirin and every other day clopidogrel) (n= 284). There were no differences in 1-year rates of death, myocardial infarction, stent thrombosis or target lesion revascularization between patients receiving a conventional compared to a modified antiplatelet regimen (11.8 vs. 11.3, respectively). There were no differences also in the rates of TIMI major bleeding between the two groups (1.1% vs. 1.4%).

Data from RIKS-HIA and SCAAR registries concerning 25091 patients treated with PCI-S in Sweden were analyzed [33]. OAC was prescribed in 1183 patients, 56% of whom were on TT. TT was associated with four times higher risk of any bleeding than OAC plus aspirin, adj. RR 4.27 (95% CI 1.2-15.1), but a lower incidence of death or MI than OAC plus clopidogrel adj. RR 0.63 (95% CI 0.40-0.99).

In a retrospective Japanese study [34], 575 consecutive patients implanted with DES were analyzed. During a median follow-up of 459 days, 14 (2.7%) patients receiving DAPT, and 9 (18%) receiving TT reported major bleeding complications (p< 0.001). These results show that adding warfarin to DAPT was associated with an increased risk of subsequent major bleeding. On the other hand, the incidence of MACE did not differ between the two groups (p= n.s.).

In a small study conducted in Barcelona [35], the authors reviewed data from consecutive patients on OAC who underwent PCI-S and were prescribed TT. Overall, hemorrhages were higher in patients receiving TT than on DAPT (90.6 and 8.29 bleedings/100 patient-years, respectively (RR 10.9; p< 0.01). Nevertheless, two patients suffered severe hemorrhages during DAPT (one traumatic subdural hematoma and one hemathemesis, both resolved uneventfully), accounting for a rate of 2.76 bleedings/100 patient-years, compared to none during TT (p= 0.4). They concluded that patients treated with PCI-S who also have indication for OAC can be prescribed a second antiplatelet drug (usually clopidogrel) with no increase in severe hemorrhages, but a significantly higher rate of non-severe hemorrhages (16-fold increased risk) that is unrelated to differences in anticoagulation control.

In a very recent publication regarding 362 Korean AF patients undergoing PCI-S [36] 20.2% of patients received TT. Globally, warfarin was prescribed to 84 patients (23.2%). Cilostazol was used in addition to dual antiplatelet therapy in 35% of the patients who did not receive warfarin. The anticoagulation group was slightly younger than the non-anticoagulation group. Overall, 45.5% of patients had a CHADS2 score of ≥2. The indications for PCI were chronic stable angina (43%) and acute coronary syndrome (57%). DESs were used in most cases. The use of warfarin did not affect the incidence of major adverse events, including death, myocardial infarction, target vessel revascularization, a bleeding episode or stroke, but it was associated with an increased risk of major bleeding (p= 0.002) by Kaplan-Meier survival analysis (Log-Rank test). Independent predictors for major bleeding were total stent number (HR 2.020) and warfarin use (HR 7.564). Authors concluded that oral anticoagulation therapy after PCI may increase hemorrhagic events in Korean AF patients.

### Consideration for clinical practice

As derived from the data above, TT appears the best antithrombotic regimen to prevent both stent thrombosis and stroke in AF patients undergoing PCI-S. As long as this regimen was carried out in fact, such complications were only exceptionally reported. In the study by Karjalainen et al. [14] however, one of the two (1.9%) episodes of stent thrombosis occurred soon after clopidogrel withdrawal. Indeed, among the patients examined by Rubboli et al. [16] the only thrombotic event (i.e., subacute stent thrombosis) was observed with the combination of warfarin and aspirin. As regards stroke prevention, the study by Karjalainen et al. [14] is again in support of TT (2.8%) and, as expected, against DAPT (8.8%). In two studies comparing TT with DAPT for more than 1 month, thromboembolic events and death were increased in patients on DAPT, while subacute stent thrombosis were not different [16,18] In the study by Gao et al. [25] whenever DAPT was used, alone or in combined with OAC, the incidence of stent thrombosis was about two times less frequent. In addition, compared with DAPT, the incidence of stroke was about four times lower in regimens including warfarin, combined to either one or two antiplatelet agents. In the study by Sambola et al. [26], in the subgroup classified at moderate to high thromboembolic risk, the overall clinical benefit of TT was evident: the incidence of thromboembolic complications was five to seven times lower than in the other groups.

The safety profile of TT is undoubtedly an issue. With the exception of a few studies [17,23,26,29,30,33,35], the overall risk of bleeding, especially major, with the combination of warfarin, aspirin, and clopidogrel was found to be relevant and higher (up to 5-fold) than with DAPT. The overall incidence of major adverse cardiovascular events and bleeding events is reported in Table 2. The absolute incidence of major hemorrhages however, appears low, at least in the short term, i.e. 1 month (about 2.5%). Administration of numerous antithrombotic drugs, such as during bridging therapy or use of glycoprotein IIb/IIIa inhibitors, seem to increase the short-term hemorrhagic risk after PCI-S [11,14,16]. Data from the GRACE registry [17] reported similar in hospital rates of major bleeding in patients with TT (5,9%) compared to warfarin plus a single antiplatelet agent (4,6%). Another study (26) reported similar rates of major and minor bleeding after 2 years follow up in patients with TT (27,5%) versus DAPT (18%). Most of the bleeding occurred in the gastrointestinal tract. In the study by Gao et al. [25], TT was characterized by a significant higher incidence of overall bleeding, even if the difference was essentially driven by minor bleeding. Notably, 72% of all the INR measurements were within the target range (1,8 - 2,5). In the by Sambola et al. study [26], TT was associated with the highest incidence of bleeding events (once again mainly minor bleeding).

**Table 2:** Incidence of MACCE and bleeding events in patients with an indication for OAC receiving TT after PCI-S

**Author**	**MACCE (%)**				**Bleeding (%)**	
	**All cause mortality**	**MI**	**Ichemic Stroke**	**Overall**	**Major**	**Overall**
Orford JL et al. [9]	NR	NR	NR	0	3.1	9.2
Mattichak SJ et al. [10]	3	29	0	NR	NR	21^¥^
Khurram Z et al. [11]	0.9	0	0	NR	6.6	21.5
Porter A et al. [12]	NR	NR	NR	NR	1	11
Lip GYH & Karpha M [13]	NR	NR	NR	NR	0	0*
Karjalainen PP et al. [14]	8.7	10	3.2	25.1	8.2	NR
DeEugenio D et al. [15]	1	NR	NR	NR	13.5	NR
Rubboli A et al. [16]	0	0	0	0	14.3	20
Nguyen MC et al. [17]	5.1	3.3	0.7	NR	5.9^§^	NR
Rogacka RCA et al. [18]	3.9	1.6	NR	23.6	4.7	7.1
Ruiz-Nodar JM et al. [19]^	17.8	6.5	1.7	26.5	14.9	27.5
M.-Fernandez S et al. [20]	5.9°	NR	NR	25.5	27.4	NR
Sarafoff N et al. [21]	10.7	3.7	1.1	14.1	1.4	9.1
Maegdefessel L et al. [22]	7.1	0	0	NR	0	NR
Rossini R et al. [23]	2	NR	1	5.8	2.9	10.8
Valencia J et al. [24]	NR	NR	NR	NR	NR	NR
Gao F et al. [25]	4.4	2.9	0.7	8.8	2.9	11.8
Sambola A et al. [26]	6.8	NR	0.3	7.9	4.3	15.5
Gilard M et al. [27]	8	5	0.8	NR	5.6	18.4
Hälg C et al. [28]	NR	NR	NR	NR	6.1	NR
Halbfass P et al. [29]	NR	NR	NR	21	8	NR
Helft G et al. [30]	0	6	0	6	0	2
Olson KL et al. [31]	14.8°	0	NR	15.4	14.3	NR
Baber J et al. [32]	5.9	1.8	NR	11.8	1.1	4.6^§^
Persson J et al. [33]	3.6	9	NR	NR	2.3	4.1
Uchidab Y et al. [34]	8	0	4	22	18	38
Guasch E et al. [35]	NR	NR	NR	NR	0	12.1
Jang SW et al. [36]	3.6	3.6	1.2	26.2	10.7	13.1

MACCE= major adverse cardiovascular and cerebrovascular events ; OAC= oral anticoagulation; TT= triple therapy;

PCI-S= percutaneous coronary intervention with stent implantation; NR= not reported.

^¥^ transfusions * requiring hospitalization ^§^ in-hospital events ^ data are related to anticoagulated patients, 88% of whom treated with TT ° cardiovascular-related death.

The meta-analysis by Zhao et al. [37] which included nine studies [10,11,14,15,19-23], demonstrated that TT is more efficacious in reducing cardiovascular events and mortality, at the price however of an increased bleeding risk. Almost all bleeding events, however, occurred in the first 6 months after discharge and were often associated with supra-therapeutic INR levels [22,23]. On the other hand, the prevalence of major bleeding increases with TT treatment duration, from 2,6% to 4,6% at 30 days, to 13,9% at 6 months, and 7,4% to 10,3% at 1 year [38]. Possible explanation for the higher bleeding events are advanced age, female gender, comorbidities like renal dysfunction and previous major bleeding, glycoprotein IIb/IIIa inhibitors use, and smoking [14]. Of note, also anemia resulted a risk marker of both mortality and hemorrhagic complications [12,15,16].

In summary, in patients with AF at moderate to high risk of stroke undergoing PCI-S, TT is generally advisable [39,40]. Careful INR monitoring is important, as well as individual bleeding risk evaluation [3,41]. Since most bleeding events appear to be gastrointestinal [10,15], gastric protection with proton-pump inhibitor should be considered in all cases on TT (such as on DAPT), being the association of a thienopyridine a risk factor for upper gastrointestinal events in low-dose aspirin users, whereas the use of proton-pump inhibitor is considered a protective factor [42]. Restrictive use of DES is recommended to keep the duration of TT as short as possible [6,38,43]. In order to reduce early hemorrhagic/access site complications, radial approach and uninterrupted anticoagulation (INR value > 2) instead of heparin bridging should be considered [3,44-48].

At present, minimal data are available on the combination of warfarin and clopidogrel, although in the small subgroup of patients receiving warfarin plus clopidogrel in the study by Karjalainen et al. [14], and in the study by Ait mokhtar et al. [49] the efficacy appeared to be good. Nevertheless, according to another study [26], the OAC plus a single antiplatelet agent was classified as having the worst profile of risk, especially in patients at moderate to high thromboembolic risk. In addition to that, the safety profile as observed in the study by Karjalainen et al. [14] appears as suboptimal as that of TT, making therefore the combination of OAC and clopidogrel a relatively attractive alternative to TT.

When a very high hemorrhagic risk is perceived, such as with a HAS-BLED (Hypertension with systolic >160 mmHg, Abnormal renal/liver function, Stroke history, Bleeding, Labile INRs, “Elderly” with age ≥65 years, Drugs/alcohol usage) [50] score ≥3, the balloon-only angioplasty strategy may be considered. Although being associated with a higher restenosis risk, avoidance of stent implantation, and consequently of long thyenopiridine use, might reduce the bleeding risk [51]. When a stent is required, placement of bare-metal stent should be generally preferred over a DES. The combination of warfarin and aspirin should not be used, owing to the suboptimal efficacy on adverse cardiac events observed both in the historical trials comparing such regimen with DAPT [7], and in the studies by Karjalainen et al. [14] and Rubboli et al. [16], where stent thrombosis and/or myocardial infarction were solely or mainly observed with that antithrombotic treatment. Treating long lesions, small vessels, diabetic patients, in-stent restenosis generally advocate the use of DES. In this case, a second-generation DES, such as everolimus or zotarolimus eluting stents, should be preferred [52] owing to the associated lower risk of thrombosis rate [53], which may likely allow for a shorter (i.e., 3 months) duration of clopidogrel (and hence TT).

### Future perspectives

Dabigatran, an oral anticoagulant from the class of the direct thrombin inhibitors, administered in two fixed doses (110 or 150 mg twice daily) in patients with non-valvular AF at moderate to high risk of stroke, demonstrated to be more effective than warfarin in reducing stroke/systemic embolism, and less likely to cause major bleeding complications [54]. Apixaban, an oral direct factor Xa inhibitor, administered at a dose of 5 mg twice daily in patients with non-valvular AF at moderate to high risk of stroke was superior to warfarin in preventing stroke/systemic embolism, reducing overall mortality and causing less bleeding [55]. Rivaroxaban, another oral factor Xa inhibitor, at the dose of 20 mg/die recently demonstrated to be non inferior to warfarin for the prevention of stroke/systemic embolism in AF patients at relatively high risk of stroke. In all three studies, the incidence of intracranial and fatal bleeding occurred less frequently with the novel oral anticoagulants [56].

The use of the oral factors Xa inhibitors rivaroxaban and apixaban in combination with DAPT, in patients with acute coronary syndrome, has been evaluated in the ATLAS ACS 2–TIMI 51 [57] and APPRAISE-2 [58] studies. In the ATLAS ACS 2-TIMI 51 study, patients were randomized to receive twice-daily doses of either 2.5 mg or 5 mg of rivaroxaban, on top of the therapy, or placebo. Rivaroxaban reduced the risk of the composite end point of death from cardiovascular causes, myocardial infarction, or stroke. Rivaroxaban increased the risk of major bleeding and intracranial hemorrhage respect to DAPT, but not the risk of fatal bleeding. In the APPRAISE-2 study patients with a recent acute coronary syndrome, and at least two additional risk factors for recurrent ischemic events, were randomized to receive apixaban, at a dose of 5 mg twice daily, or placebo, in addition to mono or dual antiplatelet therapy. The trial was terminated prematurely after recruitment of 7392 patients because of an increase in major bleeding events with apixaban (1.3% vs. 0.5%, p= 0.001), in the absence of a counterbalancing reduction in recurrent ischemic events (the primary outcome of cardiovascular death, myocardial infarction, or ischemic stroke occurred in 7.5% of patients assigned to apixaban and in 7.9% of patients assigned to placebo, p= 0.51). It would be investigated if these new anticoagulant agents can have a role in patients with AF treated with PCI-S.

On the other side, novel P2Y12 receptor antagonists, such as prasugrel and ticagrelor, showed to be superior to clopidogrel in reducing major adverse cardiovascular events [59,60], but the incidence of TIMI major bleeding was significantly higher compared to clopidogrel. Thus, at present TT with OAC, aspirin and prasugrel/ticagrelor appears not advisable owing to the likely elevated risk of bleeding.

At the same time, it seems that a key issue will be to test whether DAPT can be shortened in patients treated with the newest generations of DES, either second generation DES [61], or DES with bioresorbable polymers and fully bioresorbable stents, as the short term degradation of the polymer or of the whole scaffold should avoid per se late and very late stent thrombosis [62,63].

While waiting for further and higher quality data regarding the optimal antithrombotic regimen for AF patients requiring OAC for associated moderate to high risk of stroke and who are submitted to PCI-S, TT of warfarin, aspirin and clopidogrel with a duration as short as possible should represent the standard of care at least in patients at low to moderate risk of bleeding.

## Conclusions

In accordance with the currently available evidence, management recommendations of patients with AF undergoing PCI-S have been recently issued by experts’ panels and official Scientific bodies [1-5]. Overall, a consensus on TT is apparent. As properly acknowledged in these papers however, the current recommendations arise from limited and of relative poor quality evidence. Accordingly, most of these recommendations are graded IIa (that is, uncertain benefit over risk) with a level of evidence mostly C (that is, arising from experts’ consensus or small studies or observational registries). We suggest a practical summary of the mostly accepted recommendations, as presented in Table 3.

**Table 3:** Summary of the suggested antithrombotic strategies arising from published studies and guidelines, applied to patients with AF undergoing PCI-S

**Moderate-high TE risk (CHADS2 score** ≥ **2)**	· use radial approach	· use radial approach
	· prefer uninterrupted OAC (INR > 2)	· prefer uninterrupted OAC (INR > 2)
	· prefer BMS (DES allowed)	· consider balloon-only PCI or CABG
	· at discharge prescribe TT for 1–6 months	· prefer BMS (DES to be avoided)
	· target INR to 2.0-2.5	· at discharge prescribe TT for 2–4 weeks
	· prescribe gastric protection throughout DAPT/TT	·target INR to 2.0-2.5
		· prescribe gastric protection throughout DAPT/TT
**Low TE risk (CHADS2 score 0–1)**	· use either radial/femoral approach	· prefer radial approach
	· withdraw OAC	· withdraw OAC
	· use either BMS/DES	· prefer BMS (DES allowed, preferably last generation)
	· at discharge prescribe DAPT for 1–6 months	· at discharge prescribe DAPT for 2–4 weeks
	· prescribe gastric protection throughout DAPT	· prescribe gastric protection throughout DAPT
	**Low bleeding risk (HAS-BLED score 0–2)**	**Moderate-high bleeding risk (HAS-BLED score** ≥ **3)**

## Abbreviations

AF: Atrial fibrillation; OAC: Oral anticoagulation; VKA: Vitamin K antagonists; DAPT: Dual anti-platelet treatment; TT: Triple antithrombotic therapy; PCI-S: Percutaneous coronary intervention with stent implantation; DES: Drug eluting stent; ACS: Acute coronary syndrome; AMI: Acute myocardial infarction; ACC: American College of Cardiology; AHA: American Heart Association; ESC: European Society of Cardiology.

## Competing interests

The authors declare that they have no competing interests.

## Authors’ contributions

All authors read and approved the final manuscript.

## References

[B1] Camm AJ Kirchhof P Lip GY Guidelines for the management of atrial fibrillation. The Task Force for the Management of Atrial Fibrillation of the European Society of Cardiology (ESC) Eur Heart J 2010 31 2369 2429 20802247 10.1093/eurheartj/ehq278

[B2] Lip GY Huber K Andreotti F Management of antithrombotic therapy in atrial fibrillation patients presenting with acute coronary syndrome and/or undergoing percutaneous coronary intervention/stenting. A Consensus Document of the European Society of Cardiology Working Group on Thrombosis, endorsed by the European Heart Rhythm Association (EHRA) and the European Association of Percutaneous Cardiovascular Interventions (EAPCI) Thromb Haemost 2010 103 13 2820062939

[B3] Guyatt GH Akl EA Crowther M Antithrombotic Therapy and Prevention of Thrombosis, 9th ed: American College of Chest Physicians Evidence-Based Clinical Practice Guidelines Chest 2012 141(2)(Suppl) 7S 74S 22315257 10.1378/chest.1412S3 PMC3278060

[B4] Wijns W Kolh P Danchin N Guidelines on myocardial revascularization Eur Heart J 2010 31 2501 2555 20802248 10.1093/eurheartj/ehq277

[B5] Rubboli A Colletta M Valencia J Periprocedural Management and In-Hospital Outcome of Patients with Indication for Oral Anticoagulation Undergoing Coronary Artery Stenting J Interven Cardiol 2009 22 390 397 10.1111/j.1540-8183.2009.00468.x 19453820

[B6] Connolly S Pogue J Hart R ACTIVE Writing Group of the ACTIVE Investigators Clopidogrel plus aspirin versus oral anticoagulation for atrial fibrillation in the Atrial fibrillation Clopidogrel Trial with Irbesartan for prevention of Vascular Events (ACTIVE W): a randomised controlled trial Lancet 2006 367 1903 1912 16765759 10.1016/S0140-6736(06)68845-4

[B7] Rubboli A Milandri M Castelvetri C Cosmi B Meta-analysis of trials comparing oral anticoagulation and aspirin versus dual antiplatelet therapy after coronary stenting. Clues for the management of patients with an indication for long-term anticoagulation undergoing coronary stenting. Cardiology 2005 104 101 106 10.1159/000086918 16020950

[B8] Rubboli A Dewilde W Huber K The Management of Patients on Oral Anticoagulation Undergoing Coronary Stent Implantation: A Survey among Interventional Cardiologists from Eight European Countries J Interv Cardiol 2012 25 2 163 169 10.1111/j.1540-8183.2011.00683.x 21950351

[B9] Orford JL Fasseas P Melby S Safety and efficacy of aspirin, clopidogrel, and warfarin after coronary stent placement in patients with an indication for anticoagulation Am Heart J 2004 147 463 467 10.1016/j.ahj.2003.06.004 14999195

[B10] Mattichack SJ Reed PS Gallagher MJ Evaluation of safety of warfarin in combination with antiplatelet therapy for patients treated with coronary stents for acute myocardial infarction J Interven Cardiol 2005 18 163 166 10.1111/j.1540-8183.2005.04065.x 15966919

[B11] Khurram Z Chou E Minutello R Combination therapy with aspirin, clopidogrel and warfarin following coronary stenting is associated with a significant risk of bleeding J Invasive Cardiol 2006 18 4 162 164 16729401

[B12] Porter A Konstantino Y Iakobishvili Z Short-term triple therapy with aspirin, warfarin, and a thienopyridine among patients undergoing percutaneous coronary intervention Catheter Cardiovasc Interv 2006 68 56 61 10.1002/ccd.20733 16763985

[B13] Lip GYH Karpha M Anticoagulant and antiplatelet therapy use in patients with atrial fibrillation undergoing percutaneous coronary intervention: the need for consensus and a management guideline Chest 2006 130 1823 1827 10.1378/chest.130.6.1823 17167003

[B14] Karjalainen PP Porela P Ylitalo A Safety and efficacy of combined antiplatelet-warfarin therapy after coronary stenting Eur Heart J 2007 28 726 732 10.1093/eurheartj/ehl488 17267456

[B15] DeEugenio D Kolman L DeCaro M Risk of major bleeding with concomitant dual antiplatelet therapy after percutaneous coronary intervention in patients receiving long-term warfarin therapy Pharmacoherapy 2007 27 5 691 696 10.1592/phco.27.5.691 17461704

[B16] Rubboli A Colletta M Herzfeld J Sangiorgio P Di Pasquale G Peri-procedural and medium-term antithrombotic strategies in patients with an indication for long-term anticoagulation undergoing coronary angiography and intervention Coron Artery Dis 2007 18 193 199 10.1097/MCA.0b013e328012a964 17429293

[B17] Nguyen MC Lim YL Walton A Combining warfarin and antiplatelet therapy after coronary stenting in the Global Registry of Acute Coronary Events: is it safe and effective to use just one antiplatelet agent? Eur Heart J 2007 28 1717 1722 10.1093/eurheartj/ehm186 17562671

[B18] Rogacka R Chieffo A Michev I Dual antiplatelet therapy after percutaneous coronary intervention with stent implantation in patients taking chronic oralanticoagulation JACC Cardiovasc Interv 2008 1 1 56 61 10.1016/j.jcin.2007.11.004 19393145

[B19] Ruiz-Nodar JM Marin F Hurtado JA Anticoagulant and antiplatelet therapy use in 426 patients with atrial fibrillation undergoing percutaneous coronary intervention andstent implantation implications for bleeding riskandprognosis J Am Coll Cardiol 2008 51 8 818 825 10.1016/j.jacc.2007.11.035 18294566

[B20] Manzano-Fernandez S Pastor FJ Marin F Increased major bleeding complications related to triple antithrombotic therapy usage in patients with atrial fibrillation undergoing percutaneous coronary artery stenting Chest 2008 134 3 559 567 10.1378/chest.08-0350 18641090

[B21] Sarafoff N Ndrepepa G Mehilli J Aspirin and clopidogrelwith or without phenprocoumon after drug eluting coronary stentplacement in patients on chronic oral anticoagulation J Intern Med 2008 264 472 480 10.1111/j.1365-2796.2008.01989.x 18624903

[B22] Maegdefessel L Schlitt A Faerber J Anticoagulant and/or antiplatelet treatment in patients with atrial fibrillation after percutaneous coronary intervention. A single-center experience Med Klin (Munich) 2008 103 9 628 632 10.1007/s00063-008-1101-4 18813885

[B23] Rossini R Musumeci G Lettieri C Outcomes of long-term triple therapy with aspirin, clopidogrel, and warfarin in patientsundergoing coronary stenting [abstract] Eur Heart 2008 29 Suppl 328

[B24] Valencia J Mainar V Bordes P Observance of Antiplatelet Therapy after Stent Implantation in Patients under Chronic Oral Anticoagulant Treatment J Interven Cardiol 2008 21 218 224 10.1111/j.1540-8183.2008.00354.x 18422520

[B25] Gao F Zhou YJ Wang ZJ Comparison of Different Antithrombotic Regimens for Patients With Atrial Fibrillation Undergoing Drug-Eluting Stent Implantation Circ J 2010 74 701 708 10.1253/circj.CJ-09-0880 20208381

[B26] Sambola A Ferreira-González I Angel J Therapeutic strategies after coronary stenting in chronically anticoagulated patients: the MUSICA study Heart 2009 95 18 1483 1488 10.1136/hrt.2009.167064 19451141

[B27] Gilard M Blanchard D Helft G Antiplatelet therapy in patients with anticoagulants undergoing percutaneous coronary stenting (from STENTIng and oral antiCOagulants [STENTICO]) Am J Cardiol 2009 104 3 338 342 10.1016/j.amjcard.2009.03.053 19616664

[B28] Hälg C Brunner-La Rocca HP Kaiser C Early and late increased bleeding rates after angioplasty and stenting due to combined antiplatelet and anticoaglant therapy EuroIntervention 2009 5 425 431 10.4244/EIJV5I4A67 19755328

[B29] Halbfass P Janko S Dorwarth U Dilemma of antithrombotic therapy in anticoagulated atrial fibrillation patients squeezed between thrombosis and bleeding events: a single-center experience Europace 2009 11 957 960 10.1093/europace/eup131 19493910

[B30] Helft G Dambrin G Zaman A Percutaneous coronary intervention in anticoagulated patients via radial artery access Catheter Cardiovasc Interv 2009 73 44 47 10.1002/ccd.21758 19089936

[B31] Olson KL Delate T Johnson SG Incidence of hemorrhage among anticoagulated patients receiving antiplatelet therapy after percutaneous coronary intervention J Thromb Thrombolysis 2009 29 316 321 19407931 10.1007/s11239-009-0343-1

[B32] Baber U Akhter M Kothari S Efficacy of modified dual antiplatelet therapy combined with warfarin following percutaneous coronary intervention with drug-eluting stents J Invasive Cardiol 2010 22 80 83 20124594

[B33] Persson J Lindbaeck J Hofman-Bang C Efficacy and safety of clopidogrel after PCI with stenting in patients on oral anticoagulants with acute coronary syndrome EuroIntervention 2011 6 1046 1052 10.4244/EIJV6I9A183 21518675

[B34] Uchida Y Mori F Ogawa H Impact of anticoagulant therapy with dual antiplatelet therapy on prognosis after treatment with drug-eluting coronary stents J Cardiol 2010 55 362 369 10.1016/j.jjcc.2009.12.014 20350510

[B35] Guasch E Sionis A Reverter JC Safety issues of adjunctive clopidogrel in patients discharged after percutaneous coronary intervention with stent placement and requiring oral anticoagulation Int J Cardiol 2011 146 e1 e4 10.1016/j.ijcard.2008.12.170 19174319

[B36] Jang S-W Rho T-H Kim D-B Optimal antithrombotic strategy in patients with atrial fibrillation after coronary stent implantation Korean Circ J 2011 41 578 582 10.4070/kcj.2011.41.10.578 22125556 PMC3221899

[B37] Zhao H-J Zheng Z-T Wang Z-H “Triple Therapy” Rather Than “Triple Threat” A Meta-analysis of the Two Antithrombotic Regimens After Stent Implantation in Patients Receiving Long-term Oral Anticoagulant Treatment Chest 2011 139 2 260 270 10.1378/chest.09-3083 21285053

[B38] Rubboli A Halperin JL Antithrombotic therapy with warfarin, aspirin and clopidogrel is the recommended regime in anticoagulated patients who present with an acute coronary syndrome and/or undergo percutaneous coronary interventions Thromb Haemost 2008 100 5 752 753 18989515

[B39] Faxon DP Eikelboom JW Berger PB Consensus Document: Antithrombotic therapy in patients with atriale fibrillation undergoing coronary stenting Thromb Haemost 2011 106 571 584 10.1160/TH11-04-0262 21785808

[B40] Rubboli A Magnavacchi P Guastaroba P Antithrombotic Management and 1-Year Outcome of Patients on Oral Anticoagulation Undergoing Coronary Stent Implantation (from the Registro Regionale Angioplastiche Emilia-Romagna Registry) Am J Cardiol 2012 109 1411 1417 10.1016/j.amjcard.2012.01.353 22342850

[B41] Beyth RJ Quinn LM Landefeld CS Prospective evaluation of an index for predicting the risk of major bleeding in outpatients treated with warfarin Am J Med 1998 105 91 99 10.1016/S0002-9343(98)00198-3 9727814

[B42] Shiotani A Manabe N Kamada T Risk and preventive factors of low-dose aspirin-induced gastroduodenal injuries: A comprehensive review J Gastroenterol Hepatol 2012 27 Suppl. 3 8 12 22486865 10.1111/j.1440-1746.2012.07071.x

[B43] Lip GY Nieuwlaat R Pisters R Lane DA Crijns HJ Refining clinical risk stratification for predicting stroke and thromboembolism in atrial fibrillation using a novel risk factor-based approach: the Euro Heart Survey on atrial fibrillation Chest 2010 137 263 272 10.1378/chest.09-1584 19762550

[B44] Agostoni P Biondi-Zoccai GGL De Benedictis L Radial versus femoral approach for percutaneous coronary diagnostic and interventional procedures: systematic overview and meta-analysis of randomized trials J Am Coll Cardiol 2004 44 349 356 10.1016/j.jacc.2004.04.034 15261930

[B45] Ziakas AG Koskinas KC Gavrilidis S Radial versus femoral access for orally anticoagulated patients Catheter Cardiovasc Interv 2010 76 493 499 10.1002/ccd.22527 20882651

[B46] Jessup DB Coletti AT Muhlestein JB Elective coronary angiography and percutaneous coronary intervention during uninterrupted warfarin therapy Catheter Cardiovasc Interv 2003 60 180 184 10.1002/ccd.10595 14517922

[B47] Karjalainen P Porela P Ylitalo A Safety of percutaneous coronary intervention during uninterrupted anticoagulation treatment Int J Cardiol 2007 119 9 abstr

[B48] Lahtela H Rubboli A Schlitt A Heparin Bridging vs. Uninterrupted Oral Anticoagulation in Patients With Atrial Fibrillation Undergoing Coronary Artery Stenting Circ J 2012 76 6 1363 1368 10.1253/circj.CJ-11-1206 22447005

[B49] Bonello L Armero S Sbragia P Paganelli F Ait Mokhtar O Early and late outcomes of clopidogrel and Coumadin combination for patients on oral anticoagulants undergoing coronary stenting Cardiovasc Revasc Med 2010 11 159 162 10.1016/j.carrev.2009.05.002 20599166

[B50] Lip G Frison L Halperin JL Lane DA Comparative Validation of a Novel Risk Score for Predicting Bleeding Risk in Anticoagulated Patients With Atrial Fibrillation J Am Coll Cardiol 2011 57 173 180 10.1016/j.jacc.2010.09.024 21111555

[B51] Cantor WJ Peterson ED Popma JJ Provisional stenting strategies: systematic overview and implication for clinical decision-making J Am Coll Cardiol 2000 36 1142 1151 10.1016/S0735-1097(00)00854-8 11028463

[B52] Rubboli A Kovacic JC Mehran R Lip GY Coronary stent implantation in patients committed to long-term oral anticoagulation therapy: successfully navigating the treatment options Chest 2011 139 5 981 987 10.1378/chest.10-2719 21540213

[B53] Serruys PW Silber S Garg S Comparison of Zotarolimus-Eluting and Everolimus-Eluting Coronary Stents N Engl J Med 2010 363 136 146 10.1056/NEJMoa1004130 20554978

[B54] Connolly SJ Ezekowitz MD Yusuf S Dabigatran versus Warfarin in Patients with Atrial Fibrillation N Engl J Med 2009 361 1139 1151 10.1056/NEJMoa0905561 19717844

[B55] Connolly SJ Eikelboom J Joyne C Apixaban in Patients with Atrial Fibrillation N Engl J Med 2011 364 806 817 10.1056/NEJMoa1007432 21309657

[B56] Patel MR Mahaffey KW Garg J Rivaroxaban versus Warfarin in Nonvalvular Atrial Fibrillation N Engl J Med 2011 365 883 891 10.1056/NEJMoa1009638 21830957

[B57] Mega JL Braunwald E Wiviott SD Rivaroxaban in Patients with a Recent Acute Coronary Sindrome N Engl J Med 2012 366 9 19 10.1056/NEJMoa1112277 22077192

[B58] Alexander JH Lopes RD James S Apixaban with Antiplatelet therapy after Acute Coronary Syndrome N Engl J Med 2011 365 699 708 10.1056/NEJMoa1105819 21780946

[B59] Wiviott SD Braunwald E McCabe CH Prasugrel versus Clopidogrel in Patients with Acute Coronary Syndromes N Engl J Med 2007 57 2001 2015 17982182 10.1056/NEJMoa0706482

[B60] Wallentin L Becker RC Budaj A Ticagrelor versus Clopidogrel in Patients with Acute Coronary Syndromes N Engl J Med 2009 361 1045 1057 10.1056/NEJMoa0904327 19717846

[B61] Hahn J-Y Song YB Choi J-H Three-month dual antiplatelet therapy after implantation of zotarolimus-eluting stents: the DATE (Duration of Dual Antiplatelet Therapy AfterImplantation of Endeavor Stent) registry Circ J 2010 74 2314 2321 10.1253/circj.CJ-10-0347 20938098

[B62] Stefanini GG Kalesan B Serruys PW Long-term clinical outcomes of biodegradable polymer biolimus-eluting stents versus durable polymer sirolimus-eluting stents in patients with coronary artery disease (LEADERS): 4 year follow-up of a randomised non-inferiority trial Lancet 2011 378 1940 1948 10.1016/S0140-6736(11)61672-3 22075451

[B63] Serruys PW Ormiston J Onuma Y Absorb trial first-in-man evaluation of a bioabsorbable everolimus-eluting coronary stent system: two-year outcomes and results from multiple imaging modalities Lancet 2009 373 897 910 10.1016/S0140-6736(09)60325-1 19286089

